# Supplementary figures and images for: Nono induces Gadd45b to mediate DNA repair
Source: Life Sci Alliance. 2024 Jun 6;7(8):e202302555. doi: 10.26508/lsa.202302555 (PMC11157152; doi:10.26508/lsa.202302555)

SourceDataForFigure1

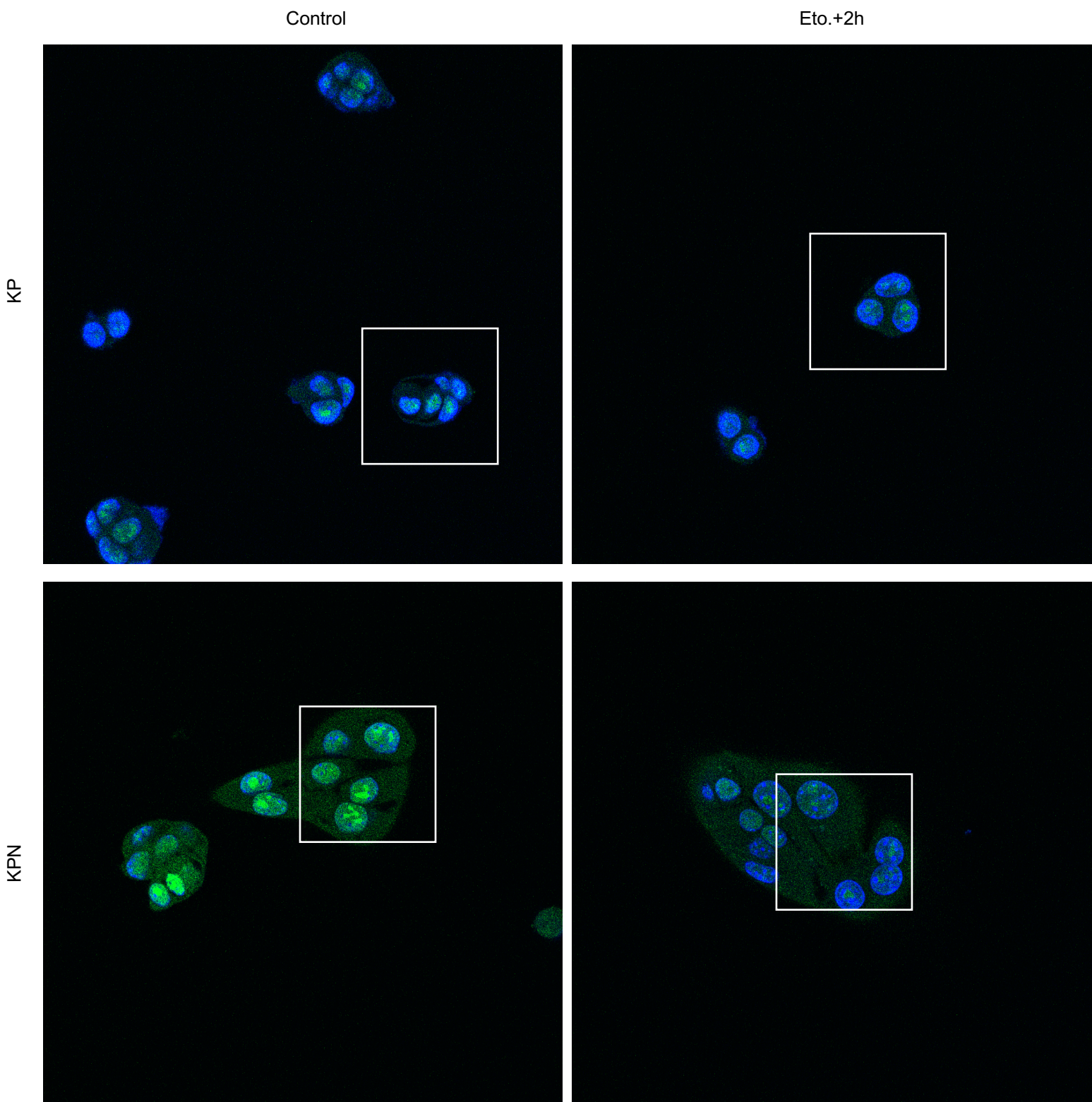

Supplement: Supplementary file 1 [file LSA-2023-02555_SdataF1.pdf]

SourceDataForFigure2

A

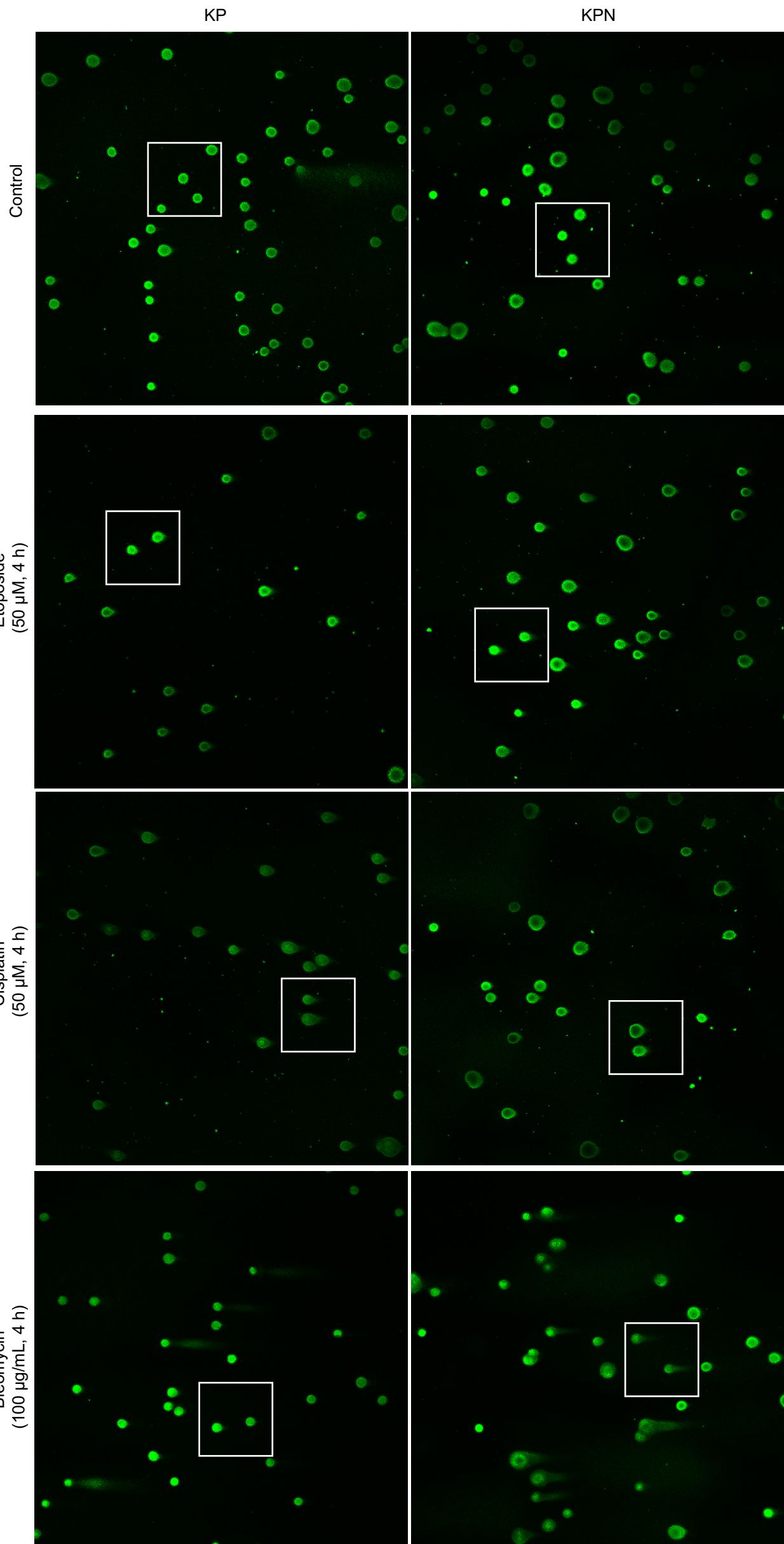

B

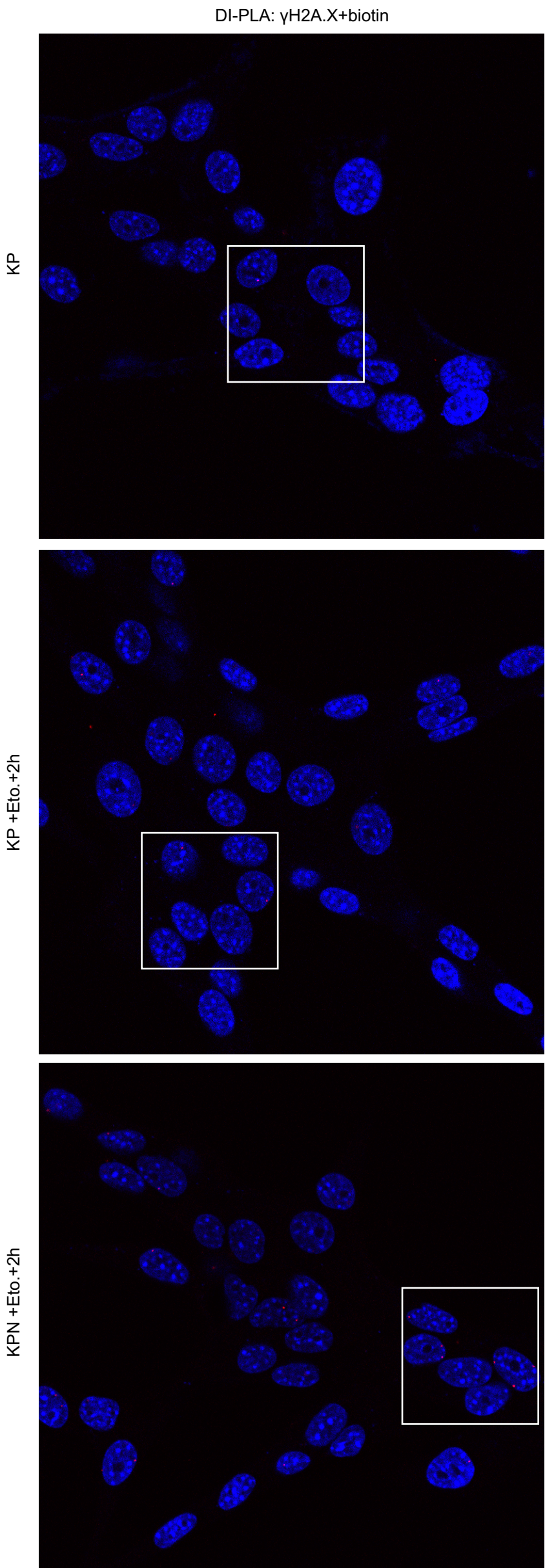

Supplement: Supplementary file 2 [file LSA-2023-02555_SdataF2.pdf]

SourceDataForFigure3

A

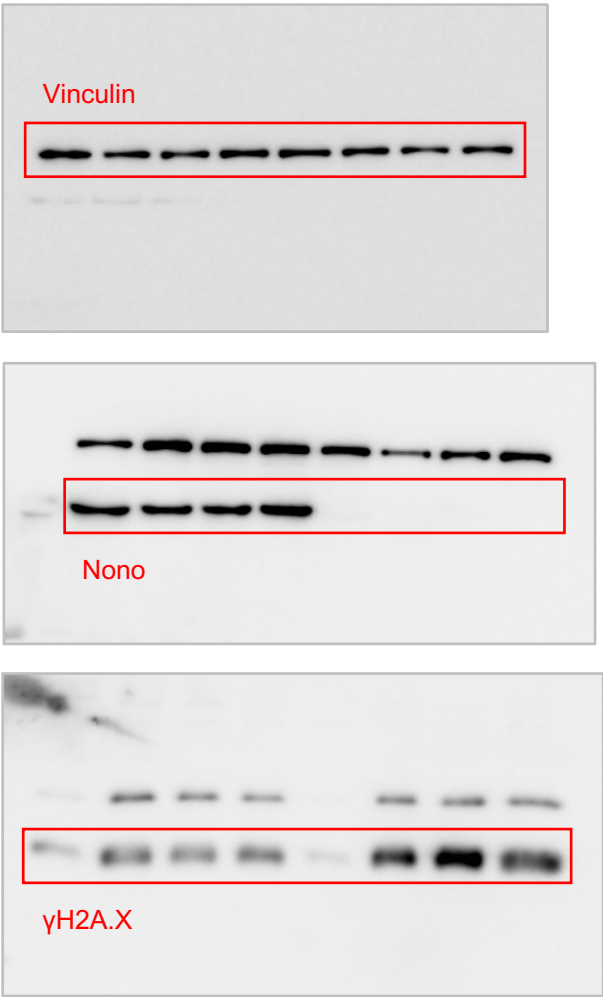

B

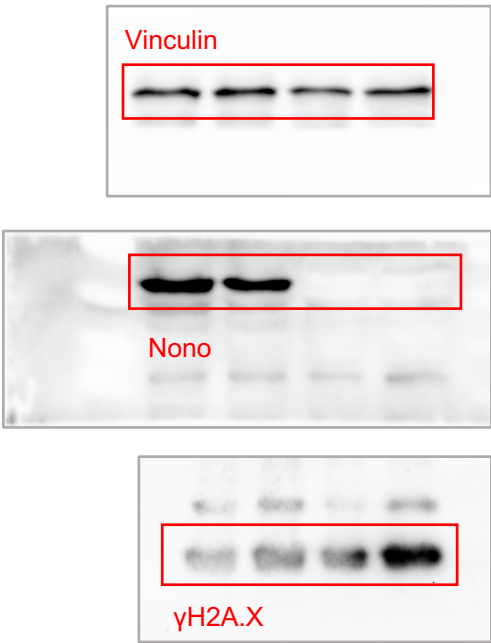

C

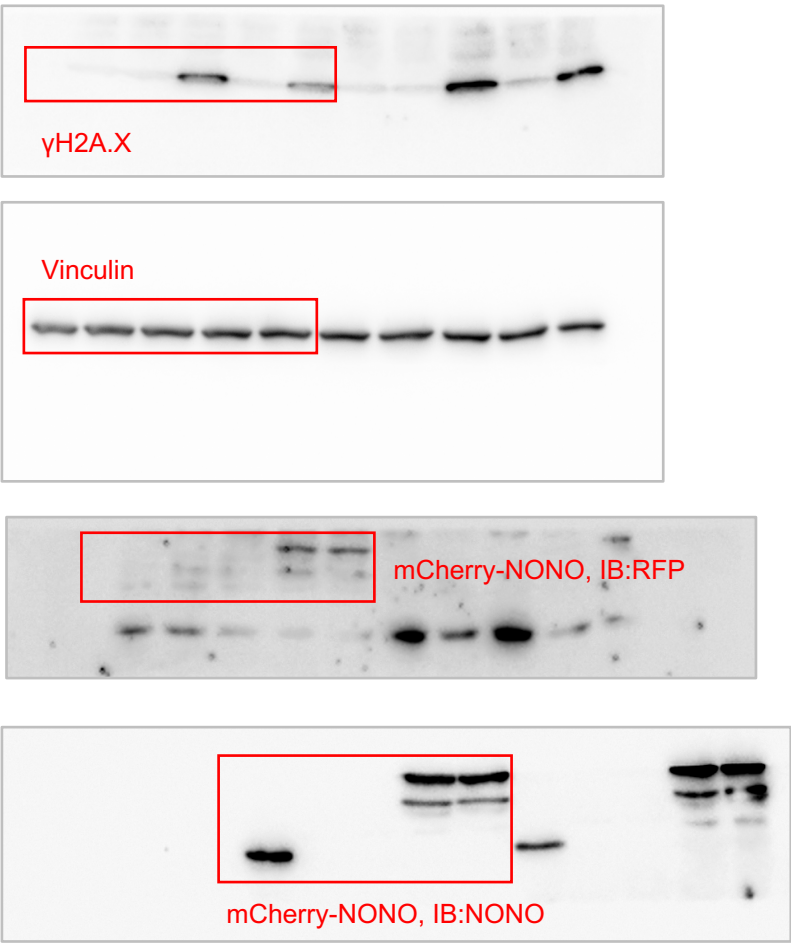

E

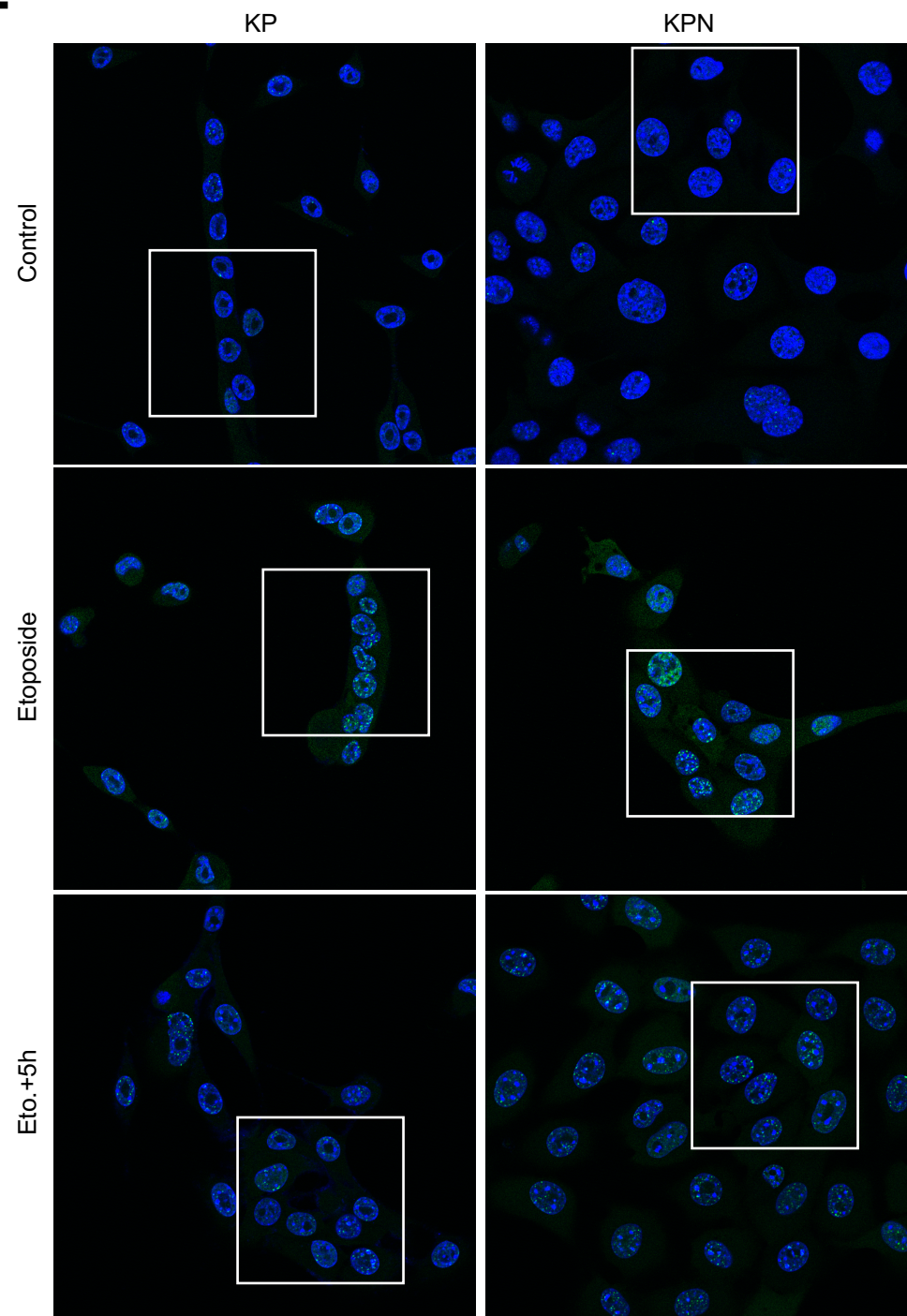

F

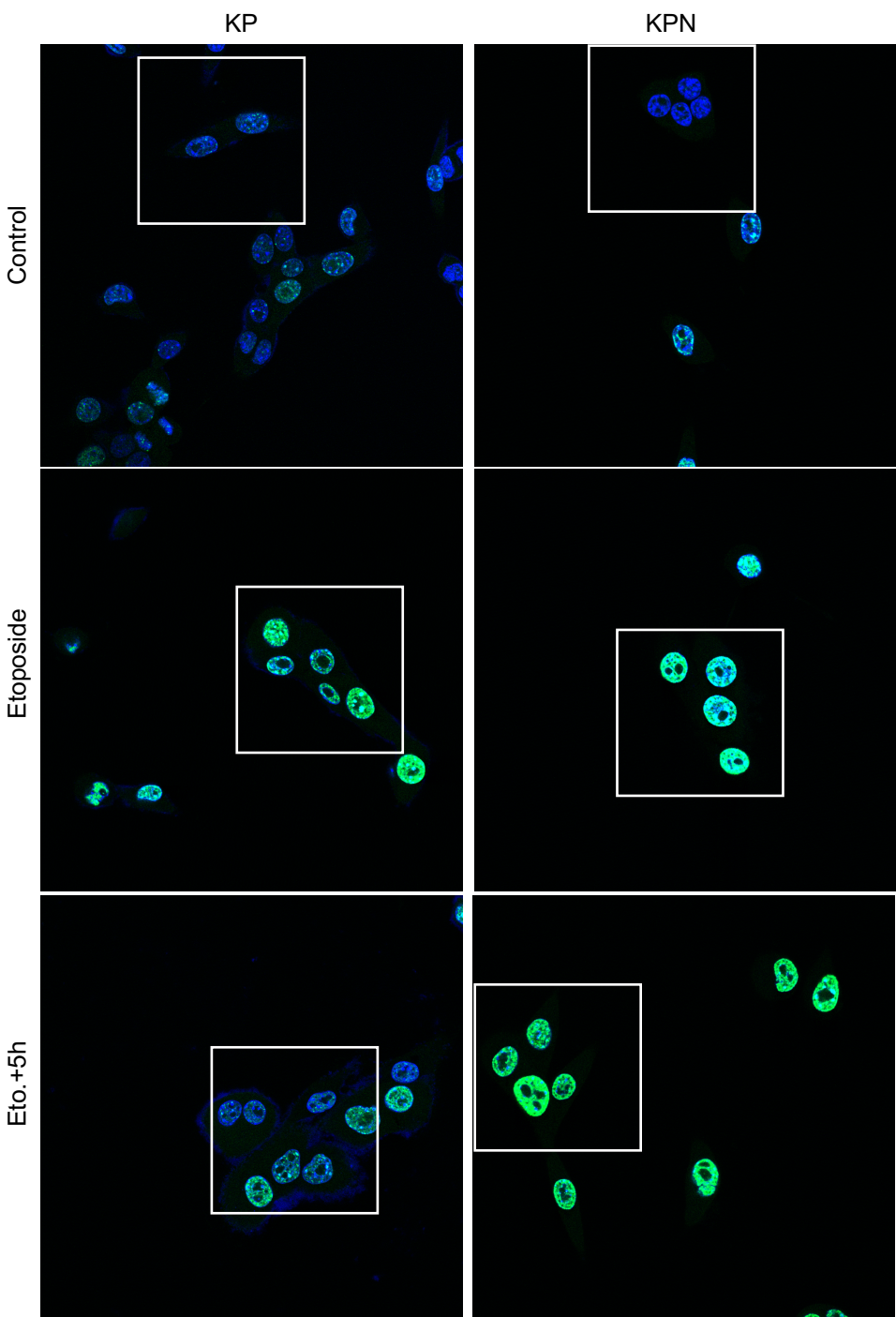

Supplement: Supplementary file 3 [file LSA-2023-02555_SdataF3.pdf]

C

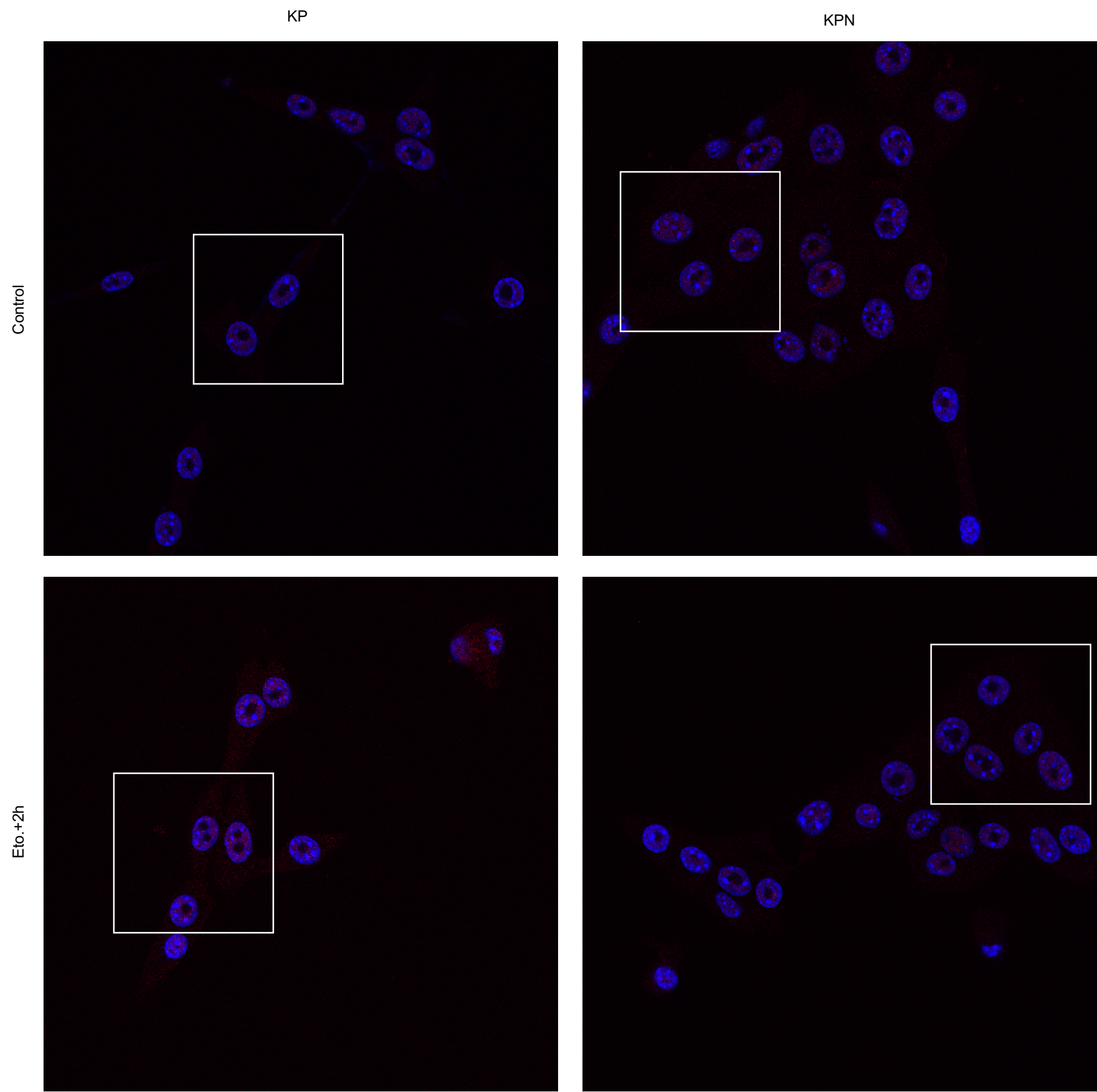

Supplement: Supplementary file 5 [file LSA-2023-02555_SdataF4.2.pdf]

# SourceDataForFigure5

A

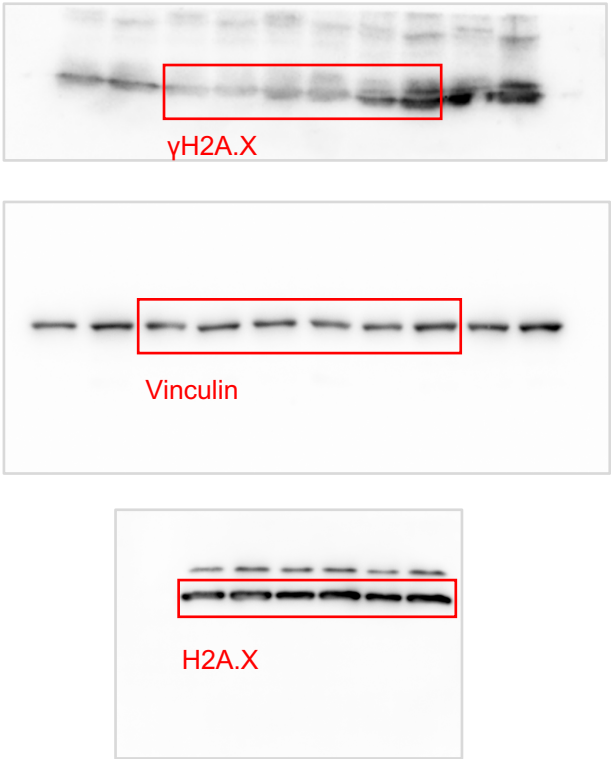

B

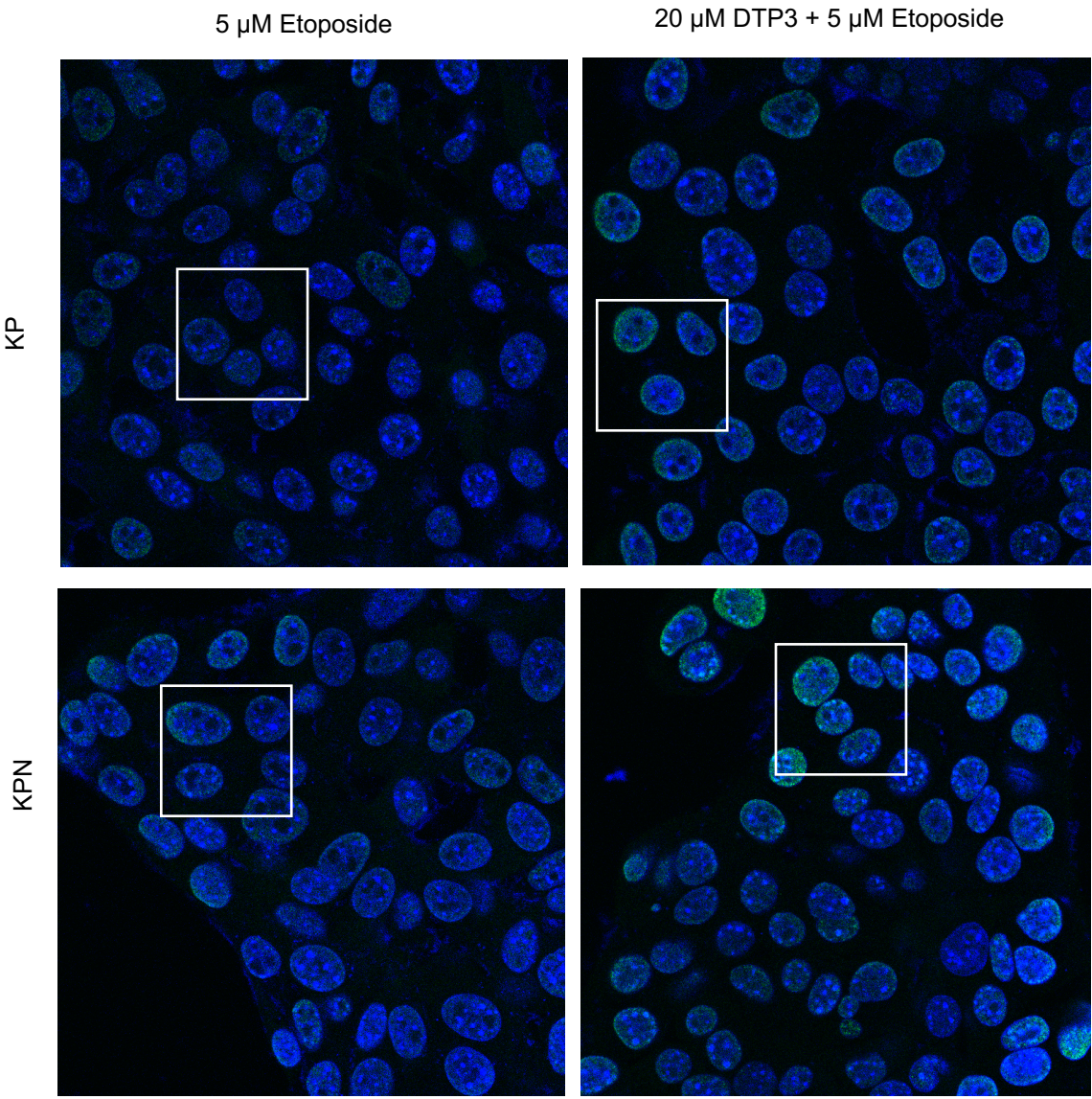

C

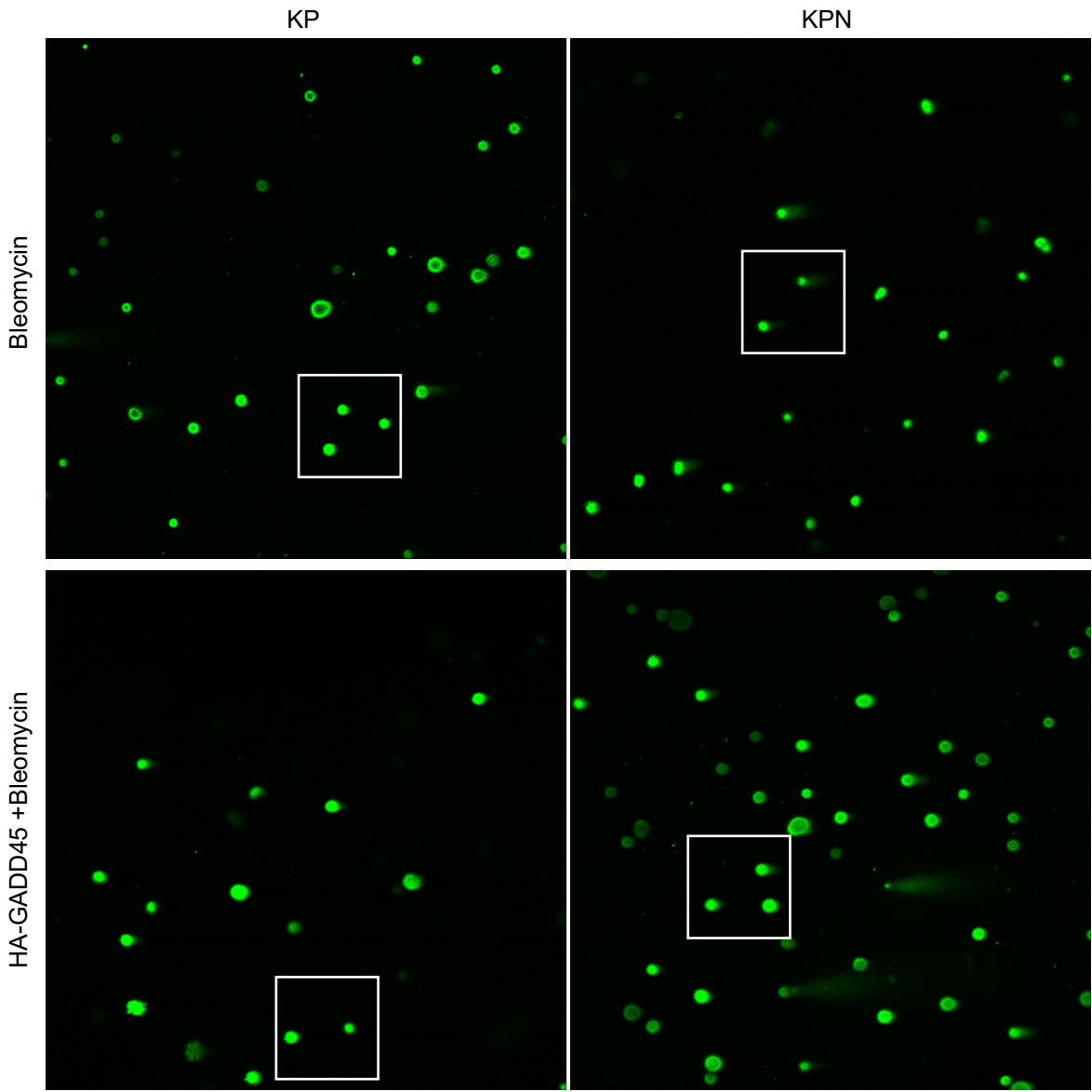

Supplement: Supplementary file 6 [file LSA-2023-02555_SdataF5.pdf]
