# Supplementary material for: Nono induces Gadd45b to mediate DNA repair
Source: Life Sci Alliance. 2024 Jun 6;7(8):e202302555. doi: 10.26508/lsa.202302555 (PMC11157152; doi:10.26508/lsa.202302555)
Supplement: Supplementary file 7 [file LSA-2023-02555_TableS1.docx]

**Table S1 -** Synthetic guide (sg)RNA DNA oligonucleotides used for CRISPR/Cas9 cloning and Nono knockout.

| **Oligonucleotide** | **Sequence (5’-3’)** |
| --- | --- |
| sgRNA-1-FWD | CACCGTACTTACGTAGTCAGCAAGA |
| sgRNA-1-REV | CATGAATGCATCAGTCGTTCTCAAA |
| sgRNA-2-FWD | CACCGATGGGAGATATACCGCATCA |
| sgRNA-2-REV | CTACCCTCTATATGGCGTAGTCAAA |
