# Supplementary material for: Nono induces Gadd45b to mediate DNA repair
Source: Life Sci Alliance. 2024 Jun 6;7(8):e202302555. doi: 10.26508/lsa.202302555 (PMC11157152; doi:10.26508/lsa.202302555)
Supplement: Supplementary file 8 [file LSA-2023-02555_TableS2.docx]

**Table S2 -** Antibodies used in this study.

| **Primary antibody** | **Species** | **Supplier, code** |
| --- | --- | --- |
| IgG control | rabbit | Proteintech, 30000-0-AP |
| Anti-Nono | rabbit | Proteintech, 11058-1-AP |
| Anti-phospho-histone H2A.X (S139) | rabbit | Cell Signaling, 2577 |
| Anti-vinculin | mouse | Sigma, V9131 |
| Anti-phospho-RNA polymerase II CTD repeat YSPTSPS (S5) | rabbit | Abcam, ab5131 |
| Anti-total-RNA polymerase II [8WG16] | rabbit | Abcam, ab26721 |
| Anti-Gadd45b | rabbit | Thermo, PA5-43160 |
| Anti-53BP1 | rabbit | Novus, NB100-304 |
| Anti-RFP tag [RF5R] | mouse | Thermo, MA5-15257 |
| Anti-biotin [BN-34] | mouse | Sigma, B7653 |
| Anti-histone H2A.X [D17A3] | rabbit | Cell Signaling, 7631 |
| Anti-HA tag | rabbit | Abcam, ab9110 |
| Anti-JNK [56G8] | rabbit | Cell Signaling, 9258 |
| Anti-phospho JNK (T183/Y185) | rabbit | Cell Signaling, 9251 |
|  |  |  |
| **Secondary antibody** | **Species** | **Supplier, code** |
| HRP-linked IgG | mouse | Cytiva, NA931 |
| HRP-linked IgG | rabbit | Cytiva, GEHENA934 |
| Alexa Fluor 488-linked IgG | rabbit | Thermo, A21206 |
| Alexa Fluor 546-linked IgG | rabbit | Thermo, A10040 |
