# Supplementary material for: Nono induces Gadd45b to mediate DNA repair
Source: Life Sci Alliance. 2024 Jun 6;7(8):e202302555. doi: 10.26508/lsa.202302555 (PMC11157152; doi:10.26508/lsa.202302555)
Supplement: Supplementary file 9 [file LSA-2023-02555_TableS3.docx]

**Table S3 -** DNA oligonucleotides used for DI-PLA. 3BiodT, biotin-tag.

| **Oligo** | **Sequence (5’-3’)** |
| --- | --- |
| FWD | TACTACCTCGAGAGTTACGCTAGGGATAACAGGGTAATATAGTTT /3BiodT/ |
| REV | TTTCTATATTACCCTGTTATCCCTAGCGTAACTCTCGAGGTAGTA |
