# Supplementary material for: Nono induces Gadd45b to mediate DNA repair
Source: Life Sci Alliance. 2024 Jun 6;7(8):e202302555. doi: 10.26508/lsa.202302555 (PMC11157152; doi:10.26508/lsa.202302555)
Supplement: Supplementary file 10 [file LSA-2023-02555_TableS4.docx]

**Table S4 -** Primer pairs used for RT-qPCR and ChIP.

| **Primer** | **Sequence (5'-3')** |
| --- | --- |
| Actb-fwd | GAGGGGAGAGGGGGTAAA |
| Actb-rev | GAAGCTGTGCTCGCGG |
| Gadd45b-fwd | CGGCCAAACTGATGAATGT |
| Gadd45b-rev | ATCTGCAGAGCGATATCATCC |
| Gapdh-fwd | AGGTCGGTGTGAACGGATTTG |
| Gapdh-rev | TGTAGACCATGTAGTTGAGGTCA |
